# Up-Regulation of miR-21, miR-25, miR-93, and miR-106b in Gastric Cancer

**DOI:** 10.29252/.22.6.367

**Published:** 2018-11

**Authors:** Pegah Larki, Alireza Ahadi, Ali zare, Shahriar Tarighi, Mahrokh Zaheri, Mojgan Souri, Mohammad Reza Zali, Hamid Ghaedi, Mir Davood Omrani

**Affiliations:** 1 Department of Medical Genetics, School of Medicine, Shahid Beheshti University of Medical Sciences, Tehran, Iran; 2 Department of Medical Laboratory Sciences, School of Allied Medical Sciences, Shahid Beheshti University of Medical Sciences, Tehran, Iran; 3 Gastroenterology and Liver Diseases Research Center, Research Institute for Gastroenterology and Liver Diseases, Shahid Beheshti University of Medical Sciences, Tehran, Iran; 4 Urogenital Stem Cell Research Center, Shahid Beheshti University of Medical Sciences, Tehran, Iran

**Keywords:** Biomarkers, microRNAs, Stomach cancer

## Abstract

**Background:**

Differential expression profile of microRNAs (miRNAs) could be a diagnosis signature for monitoring gastric cancer (GC) progression. In this study, we focus on the comparison of expression levels of miR-21, miR-25, miR-93, miR-106b, and miR-375 during the sequential pattern of GC development, including normal gastric, gastric dysplasia, and GC sample.

**Methods:**

We used SYBR Green-based quantitative-PCR to quantify miRNAs expression.

**Results:**

Our analysis revealed the increased expression levels of miR-21 (*p* = 0.034), miR-25 (*p* = 0.0003), miR-93 (*p* = 0.0406), and miR-106b (*p* = 0.023) in GC samples. In addition, GC patients with positive lymph node metastasis showed the up-regulation of miR-25, miR-93, and miR-106b (*p* < 0.05).

**Conclusion:**

Our findings suggested that the expression of miR-21, miR-25, miR-93, and miR-106b altered in GC, and some of them may be further investigated as biomarkers for GC early detection and prognosis prediction.

## INTRODUCTION

Gastric cancer (GC) is the fourth most prevalent cancer in the world, accounting for the second leading cause of cancer-related mortality[1,2]. Approximately, one million new cases of GC are estimated to occur per year; however, the incidence of GC is relatively higher in Asian than Western countries[2-4]. Risk factors for GC include sex (male to female ratio 2:1), environmental factors (*Helicobacter pylori* infection, nutrition, smoking, and obesity), blood group A, genetic factors (mutation in *P53*, *MCC*, and *APC*) and epigenetic factors (dysregulation of gene expression in GC)[5]. GC diagnosis in its early stages helps to achieve more successful treatment, but its diagnosis is very difficult for patients with no apparent clinical features. The lack of specific diagnostic biomarkers in the early stages of GC is another problem for the diagnosis and treatment of GC[6]. Unfortunately, GC is diagnosed mostly at advanced stages accompanied by extensive invasion and lymphatic metastasis. Patients with advanced GC tend to develop recurrent diseases, exhibiting poor survival rates. Thus, early detection of GC is crucial to reduce mortality rates and improve the prognosis. In addition, there is a need to develop accurate and versatile diagnostic biomarkers capable of diagnosing GC at various stages[7]. Typically, tumor markers, such as CEA, CA19-9, CA125, and CA 72-4, are commonly used to detect GC; however, such markers suffer from low sensitivity and specificity, highlighting their deficiency in the early diagnosis of GC[8,9].

microRNAs, also known as miRNAs, are small, endogenous non-coding RNA molecules consisting of approximately 22 nucleotides. miRNAs, found in plants, animals, and some viruses, control gene expression through RNA silencing and post-transcriptional regulation[10]. Importantly, miRNAs regulate gene expression via either translational suppression or mRNA degradation[11-14]. miRNAs play an important role in regulating gene expression in tumorigenesis, suggesting their promising potential as diagnostic markers for malignancies. Over the past few years, a variety of studies have demonstrated miRNA expression deregulation in GC development, especially in normal versus tumor samples. Of note, hsa-miR-21, hsa-miR-25, hsa-miR-93, hsa-miR-106b, and hsa-miR-375 are commonly expressed in GC, which are deregulated in GC compared with normal tissues[10,15-18].

We hypothesized that there are unique miRNA expression profiles capable of distinguishing normal gastric tissue (NG), gastric dysplasia (GD), and GC. Furthermore, because miRNA expression is associated with cell differentiation, specific miRNAs may become deregulated in transition from NG tissue to dysplasia and GC. Identification of such differences in miRNA expression can discriminate patients who have a high risk of GC or who need to be followed carefully or treated quickly. The present study set out to identify an ideal biomarker abnormally expressed in the pre-cancerous lesion, normal tissue, and GC.

The selection of miRNAs was based on previous studies[10,15-18] on miRNA profiling of cancerous tissues. Accordingly, in this study, we examined the expression of miR-21, miR-25, miR-93, miR-106b, and miR-375 in the Iranian population.

## MATERIALS AND METHODS

### Clinical samples

Tissue biopsy samples included in this study were retrospectively selected from cases at the Research Institute for Gastroenterology and Liver Diseases (RIGLD) tissue bank (Tehran, Iran) between 2015 and 2017, with ethical approvals from the organization Ethics Committee (IR.SBMU.MSP.REC.1395.590). Formalin-fixed, paraffin-embedded (FFPE) blocks were available for 29, 33, and 39 cases of NG, GD, and GC, respectively. Demographic data and information on the clinical history and pathologic findings were documented.

### RNA extraction

Four to five thick (10 μm) sections of FFPE tissue were used to extract total RNA. For the analysis of GD and GC samples, a pathologist assessed the slides to ensure the appropriate selection of tumor tissue and blocks with more than 50% tumor content per block. Deparaffinization solution (Qiagen, Hilden, Germany) was used to remove paraffin from FFPE tissue. For RNA extraction, we used the Qiagen miRNeasy FFPE kit (Qiagen) according to the manufacturer’s protocol. Total RNA concentration was determined by measuring the ratio of the absorbance at 260 and 280 nm using a NanoDrop™ 2000c Spectrophotometer (Thermo Fisher Scientific, USA). Total extracted RNA was stored at -70 °C until use.

### miRNA quantitation

Quantitative real-time PCR (qRT-PCR) was applied to analyze miRNA expression. Briefly, the poly (A) tail was added to the extracted RNA by using the Poly (A) Tailing kit (New England Biolabs GmbH, Germany) at 37 °C for 30 min as per manufacturer’s protocol. cDNA was synthesized using miScript II RT kit (Qiagen) and then diluted 1:5. The diluted cDNA sample (two µl) was used as a template for qRT-PCR by SYBR Premix Ex TaqTM II (TaKaRa, Japan). Two µl of template cDNA was mixed with 12.5 µl of 2× SYBR Green PCR master mix and 1 µl of miR-specific forward and universal reverse primer (Table 1) in a final volume of 25 µl. The PCR was performed in duplicate, according to the standard program on Rotor-Gene Q instrument (Qiagen) as follows: 10 min at 95 °C, followed by three cycles of amplification (15 s at 94 °C and 30 s at 60 °C) and finally a dissociation curve step (ramp from 60 to 95 °C) to verify amplification specificity. All the melting curves contained single peaks, indicating specific PCR amplification. Moreover, the PCR product size was tested by agarose gel electrophoresis. PCR efficiency was evaluated by LinRegPCR software (http://www.hartfaalcentrum.nl). Afterwards, the expression level of miRNA was determined using 2^−ΔΔCt^ and normalized to U6 small nuclear RNA[19].

**Table 1:** List of the candidates and primers used in this study

Names	Sequence
Universal reverse primer	GCGAGCACAGAATTAATACGACTC
miR-375	TTTATTCGTTCGGCTCGCGT
miR-21	GGGGTAGCTTATCAGACTGATGTT
miR-25	ACATTGCACTTGTCTCGGTCT
miR-106b	CGGTAAAGTGCTGACATTGCA
miR-93	CAAAGTGCTGTTCGTGCAGGT
U6	CGCAAGGATGACACGCAAATTC

### Statistical analysis

Student’s *t*-test and ANOVA were used to analyze the expression levels of miRs (miR-21, miR-25, miR-93, miR-106b, and miR-375) between two and three groups. In case of non-parametric data distribution, non-parametric tests (Mann-Whitney U and Kruskal-Wallis) were applied. Statistical analysis was performed using SPSS 16 (SPSS, Inc., Chicago, IL, USA). *p* values less than 0.05 were considered to be statistically significant. The sensitivity and specificity of each miRNA between normal and GC tissue as well as area under curve (AUC) and Youden index were calculated using the MedCalc statistical software 15 (MedCalc Software, Ostend, Belgium).

## RESULTS

Our result evidenced no significant differences for the mean of age in NG, GD, and GC (52.03 ± 15.9, 64.32 ± 14.35, and 62.38 ± 13.47, respectively). The male to female sex ratios were found to be 1.23, 3.12, and 1.43 in NG, GD, and GC, respectively.

### Identification of elevated miRNAs among normal, dysplastic, and cancerous tissues

**Fig. 1:**
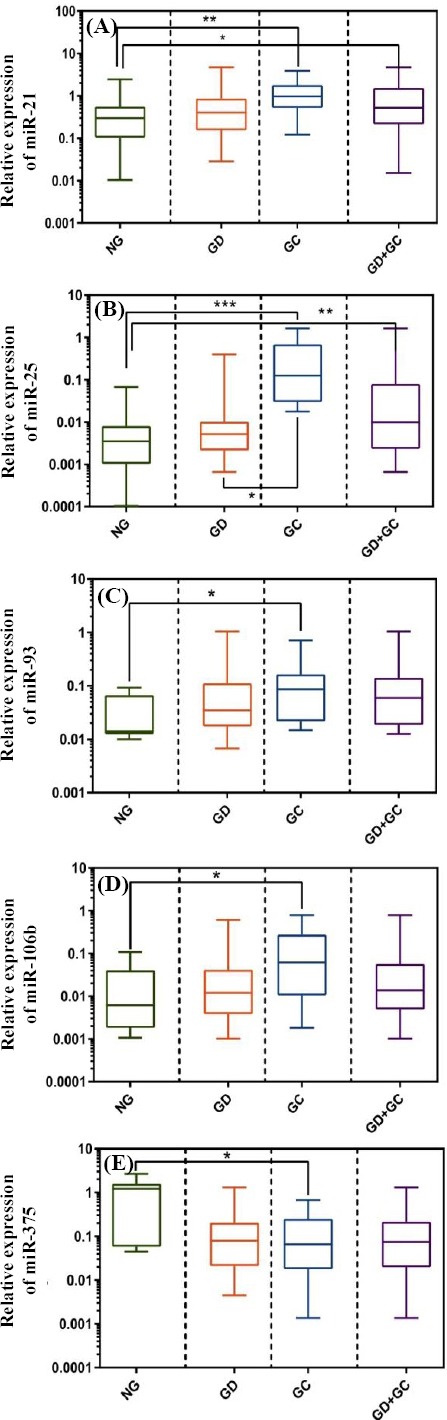
Relative expression (2^-ΔΔCt^) of miR-21 (A), miR-25 (B), miR-93 (C), miR-106b (D), and miR-375 (E) in the studied samples. ^*^, ^**^, ^***^ indicate *p* ≤ 0.05, *p* ≤ 0.01, and *p* ≤ 0.001, respectively.

Figure 1 shows a significant up-regulation in miR-21 (fold change: 2.53) and miR-25 (fold chage: 2.94) in GC compared to NG (*p* < 0.00). In addition, miR-25 expression showed a significant up-regulation in GC compared with NG (*p* = 0.03). As shown in Figure 1A-1D, the expression levels of miR-93, miR-106b, and miR-25 were significantly up-regulated in GC compared to normal tissue (*p* < 0.05). We found no significant dysregulation of miR-375 in GC compared to NG and GD (Fig. 1E and Table 2).

**Table 2:** ΔCT of each miRNA in three groups (GC, GD, and NG) and between GC and GD groups

miRNA	GC (n = 39)	GD (n = 33)	NG (n = 29)	*p* value	GC + GD (n = 72)	NG	*p* value
miR-21	2.75±1.068	3.3±2.26	3.78±1.78	0.0048	2.65±1.21	3.78±1.78	0.0175
miR-25	5.11±4.24	5.96±2.54	4.25±3.29	0.0003	6.08±4.38	4.25±3.29	0.035
miR-93	5.083±2.23	6.52±2.34	3.47±2.75	0.0518	6.23±3.28	3.47±2.75	0.124
miR-106b	6.127±4.123	9.02±4.22	9.76±5.43	0.0035	5.05±4.08	9.76±5.43	0.0528
miR-375	4.63±4.06	3.70±3.28	3.68±1.57	0.3093	4.15±3.66	3.68±1.57	0.72

One-way ANOVA test was used to calculate the mean ± SD of miRNA in each group by multiple-comparison, and also the *t*-test was conducted for analysis between two (GC + NG and NG) groups.

### Clinical characteristics of the study subjects

The demographic and clinicopathological data for various miRNAs were retrospectively obtained from patients’ medical records, as depicted in Table 3. As evident from the results of Mann-Whitney test, the expression profile of candidate miRNAs was significantly correlated with several factors, including TNM Classification of Malignant Tumors (TNM) stage, lymph node metastases, and *H. pylori* infection (*p* < 0.05).

**Table 3:** Comparison between candidate miRNA expression rates (mean of ΔCt ± SD) according to the clinicopathological characteristics of GC patients

Variable	N	miR-21		miR-25		miR-93		miR-106b	
			
		Mean ± SD	*P* value	Mean ± SD	*P* value	Mean ± SD	*P* value	Mean ± SD	*P* value
Age									
>50	64	3.20 ±2.76	0.0893	4.175 ± 3.166	0.6344	6.36 ± 5.176	0.0581	6.35 ±5.08	0.175
<50	50	5.79 ±4.25		5.46 ± 5.038		6.32 ± 5.32		6.32 ± 5.34	
Sex									
Male	65	4.750 ± 3.166	0.6654	5.33 ± 4.38	0.270	6.45 ± 5.031	0.270	6.886 ± 6.36	0.270
Female	49	5.038 ± 5.46		5.91 ± 4.16		5.991± 4.754		3.195 ±2.98	
TNM									
I, II	14	1.048 ± 1.009	0.0309	5.59 ± 3.38	0.0253	4.171 ± 3.521	0.5830	8.137 ± 7.93	0.0138
III, IV	20	5.036 ± 4.070		3.280 ±2.524		4.843 ± 3.524		4.62 ±3.905	
LNM									
Positive	18	4.62 ± 4.43	0.0259	4.62 ±4.43	0.0439	6.848 ±5.094	0.0402	3.21 ± 1.32	0.0070
Negative	20	4.38 ± 2.635		4.38 ±2.63		3.427 ±2.327		3.169± 2.138	
*H. Pylori*									
Positive	64	4.835 ± 2.935	0.0014	3.624 ± 3.28	0.0128	4.865 ± 4.524	0.2313	7.34 ±6.92	0.0023
Negative	32	1.635 ± 1.122		6.134 ± 5.35		3.427 ± 2.124		6.21 ± 4.724	

### miRNAs expression profile for discrimination of GC from normal tissues

**Fig. 2:**
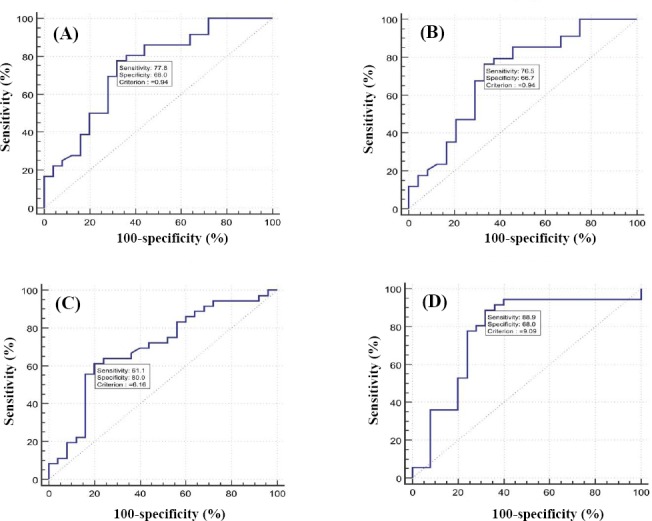
ROC curve analysis using different miRNAs for discriminating GC from normal tissue. ROC curves were constructed to show the specificity and sensitivity of miR-21 (A), miR-25 (B), miR-93 (C), and miR-106b (D).

The ROC curve analysis was performed on all the miRNAs to investigate the performance of the five mentioned miRNAs as a discriminatory tool to classify tissues in GC, GD, and normal groups. As shown in Figure 2 and Table 4, the ROC curves of the candidate miRNAs demonstrate that these miRNAs could discriminate between cancerous and non-cancer tissue with a relatively high sensitivity and specificity. Besides, the sensitivity and specificity for miR-21 were detected to be 77.08% and 68%, respectively, with an AUC of 0.74 (95% CI = 0.61–0.84, *p* < 0.00), based on the data presented in Figure 2A. The data presented in Figure 2B demonstrates that miR-25 alone could achieve a suitable diagnosis accuracy to distinguish GC tissue from non-cancerous tissue, with sensitivity of 72.2%, specificity of 100%, and AUC of 0.91 (95% CI = 0.81–0.97; *p* < 0.00). Moreover, we found that the expression profiles of miR-21, miR-25, miR-93, and miR-106b together have a specificity of 80.08% and sensitivity of 48.30% to distinguish between GC tissue from non-cancer normal, with an AUC of 0.674 (95% CI = 0.611-0.733, *p* < 0.0001.

**Table 4:** ROC curves test for determination of specificity, sensitivity, and AUC for candidates miRNA

miRNA	AUC	Specificity	Sensitivity	*P* value	Youden index	Cut-off (Δct)
miR-21	0.742	68	77.8	0.0003	0.4578	0.94
miR-25	0.726	66.7	76.5	0.0125	0.4314	0.94
miR-93	0.698	80	61	0.0049	0.4111	6.16
miR-106b	0.771	68	88.9	0.0001	0.5689	9.09

## DISCUSSION

In recent years, a wide variety of studies have focused on the role of miRNAs, as promising biomarkers, in the diagnosis and screening of cancers at early stage[20-23]. Unlike classical biomarkers, miRNAs exhibit a lot of advantages, including high stability, low degradation, and tissue-specific properties[24]. miRNAs are aberrantly expressed in malignant in comparison with normal tissues, indicating the importance of miRNA roles in tumor formation. Importantly, the differential expression patterns of miRNAs may be a useful alternative for both tumor classification and early diagnosis. Additionally, miRNA expression has been exhibited to correlate not only with different cancer types but also with cancer progression[25]. In the present study, we assessed five miRNAs, including miR-21, miR-25, miR-93, miR-106b, and miR-375, followed by qRT-PCR analysis. Our findings revealed that these miRNAs can be used as potentially suitable biomarkers for detecting and distinguishing cancer from non-cancer tissues. Our data also indicated that miR-21, miR-25, miR-93, and miR-106b had a higher expression in the GC tissue when compared with normal tissue. Moreover, pre-cancer tissue (dysplasia) and cancer tissue showed a significant difference in the expression levels of miR-25 (*p* < 0.05). This implies that miR-25 expression levels can be used to diagnose GC patients at early stage because of the multi-step processes of the gastric carcinogenesis[26]. In parallel, recent studies have suggested that miR-21 can be used as a biomarker for early diagnosis of GC. Shiotani *et al*.[27] have introduced miR-21 as a potential biomarker and cancer tissue showed a significant difference in the expression levels of miR-25 (*p* < 0.05). More importantly, miR-25 can be used to diagnose GC for the diagnosis of *H. pylori*-infected GC patients. *Cui et al*.[28] have discovered that miR-21 expression levels in GC patients are significantly higher than those in normal individuals. In fact, miR-21 up-regulation can change the biological process of cancer cells, including proliferation, apoptosis, and cellular invasion, probably via regulating *RECK* and *PTEN* as the main target genes. Zhang *et al*.[29] have found a reverse correlation between the expression level of miR-21 and *RECK* gene, so that the increased expression of miR-21 leads to the decreased expression of *RECK*. It has been suggested that the down-regulation of *RECK* plays a role in the progression of GC. Previous investigations have revealed that *FOXO3*, one of the key target genes of miR-25, critically act as a transcription factor in autophagy processes, cell cycle progression, and apoptosis[30,31].

miR-106b and miR-93 belong to the miR-106b-25 cluster, which resides in the 13^th^ intron of the DNA replication gene MCM7 on chromosome 7 in human beings[32]. These two miRNAs have been found to be highly expressed in many cancers, particularly in GC[33,34]. A study carried out by Petrocca *et al*.[35] have suggested the oncogenic role of the miR-106b/miR-93 cluster in the progression of GC through the up-regulation effect of these two miRNAs on tumor-suppressor target genes such as P21 and BIM.

Our results indicated that the overexpression of miR-21, miR-25, and miR-106b are closely correlated with the TNM stage (*p* < 0.05), *H. pylori* infection (*p* < 0.05), and lymph node metastases (*p* < 0.05), which are the main prognostic factors for GC.

In a study conducted in 2011, Matsushima *et al*.[36] demonstrated that there were significant differences in the miRNA expression signature between *H. pylori*-infected and *H. pylori*-uninfected gastric mucosa. Considering the role of *H. pylori* in chronic inflammation in gastric mucosa, this difference in miRNA expression can be applied as a prognostic factor. Based on recent reports, the expression of miRNAs has a specific relationship with lymph node metastasis, which is a prognostic factor for GC patients[7,23].

In conclusion, given the significant differences in the expression of candidate miRNAs, especially miR-25, in cancer samples compared with normal tissue, as well as their correlation with clinicopathological characteristics that play an important role in the development and progression of GC, these miRNAs can be considered as a suitable biomarker for GC early diagnosis. However, further studies are required to determine their certain roles in GC diagnosis.
